# A multi-center field study of two point-of-care tests for circulating *Wuchereria bancrofti* antigenemia in Africa

**DOI:** 10.1371/journal.pntd.0005703

**Published:** 2017-09-11

**Authors:** Cédric B. Chesnais, Naomi-Pitchouna Awaca-Uvon, Fatoma K. Bolay, Michel Boussinesq, Peter U. Fischer, Lincoln Gankpala, Aboulaye Meite, François Missamou, Sébastien D. Pion, Gary J. Weil

**Affiliations:** 1 IRD UMI 233-INSERM U1175-Montpellier University, Montpellier, France; 2 National Onchocerciasis Control Programme, Ministry of Public Health, Kinshasa, Democratic Republic of the Congo; 3 Liberian Institute for Biomedical Research, Charlesville, Liberia; 4 Infectious Disease Division, Department of Medicine, Washington University School of Medicine, Saint Louis, Missouri, United States of America; 5 Ministry of Health and Social Welfare of Côte d’Ivoire, Abidjan, Côte d'Ivoire; 6 Programme National de Lutte contre l’Onchocercose, Ministère de la Santé et de la Population, Brazzaville, Republic of Congo; The University of Kansas, UNITED STATES

## Abstract

**Background:**

The Global Programme to Eliminate Lymphatic Filariasis uses point-of-care tests for circulating filarial antigenemia (CFA) to map endemic areas and for monitoring and evaluating the success of mass drug administration (MDA) programs. We compared the performance of the reference BinaxNOW Filariasis card test (ICT, introduced in 1997) with the Alere Filariasis Test Strip (FTS, introduced in 2013) in 5 endemic study sites in Africa.

**Methodology:**

The tests were compared prior to MDA in two study sites (Congo and Côte d'Ivoire) and in three sites that had received MDA (DRC and 2 sites in Liberia). Data were analyzed with regard to % positivity, % agreement, and heterogeneity. Models evaluated potential effects of age, gender, and blood microfilaria (Mf) counts in individuals and effects of endemicity and history of MDA at the village level as potential factors linked to higher sensitivity of the FTS. Lastly, we assessed relationships between CFA scores and Mf in pre- and post-MDA settings.

**Principal findings:**

Paired test results were available for 3,682 individuals. Antigenemia rates were 8% and 22% higher by FTS than by ICT in pre-MDA and in post-MDA sites, respectively. FTS/ICT ratios were higher in areas with low infection rates. The probability of having microfilaremia was much higher in persons with CFA scores >1 in untreated areas. However, this was not true in post-MDA settings.

**Conclusions/Significance:**

This study has provided extensive new information on the performance of the FTS compared to ICT in Africa and it has confirmed the increased sensitivity of FTS reported in prior studies. Variability in FTS/ICT was related in part to endemicity level, history of MDA, and perhaps to the medications used for MDA. These results suggest that FTS should be superior to ICT for mapping, for transmission assessment surveys, and for post-MDA surveillance.

## Author summary

The Global Programme to Eliminate Lymphatic Filariasis (LF) uses point-of-care tests for circulating filarial antigenemia to map endemic areas and for monitoring and evaluating the success of mass drug administration (MDA) programs. We compared the performance of the reference BinaxNOW Filariasis card test (ICT) with the Alere Filariasis Test Strip (FTS, introduced in 2013) in 3,682 people in 5 endemic study sites in Africa. Our study confirmed the increased sensitivity of FTS reported in prior studies. FTS/ICT ratios tended to be higher in areas with low residual endemicity after MDA. However, FTS rates were only slightly higher than ICT rates in young children. These results suggest that FTS should be superior to ICT as a tool for mapping and for monitoring and evaluating LF elimination programmes.

## Introduction

Lymphatic filariasis (LF) is a major neglected tropical disease (NTD) that the World Health Organization (WHO) has targeted for elimination by 2020 [1]. The Global Programme to Eliminate Lymphatic Filariasis (GPELF, launched in 2000) uses repeated rounds of mass drug administration (MDA) with antifilarial drugs to interrupt transmission of the parasite. In 2015, MDA programs were active in 45 countries, and plans were being made to launch MDA in 6 other countries [2].

The first point of care (POC) test for circulating filarial antigenemia (CFA) was introduced in the late 1990s, and antigen tests have been used in GPELF since it started in 2000. CFA tests detect a 200 kDa *Wuchereria bancrofti* adult worm product [3]. Antigen testing is more sensitive for filarial infection than tests that detect microfilariae (Mf) in night blood. POC antigen tests can be performed with finger prick blood collected during the day or night, and they provide a visual result in 10 minutes [4,5]. They include the BinaxNOW Filariasis card test (Alere, Scarborough, ME) [6], which is an immunochromatographic card test (ICT) used by the GPELF since 2000, and the Alere Filariasis Test Strip (FTS) that was introduced in 2013. ICT card tests have been very useful for LF elimination programs, because they can be used to test people during the day or night while Mf testing requires night blood testing in most endemic areas. However, in settings in the tropics with limited resources, the FTS has several important advantages over the ICT. It is less costly, it has a longer shelf life, and it has greater analytical sensitivity (a lower limit of detection) [7].

ICT card tests or FTS are widely used during each step of LF elimination programs [8]. First, the tests can be used for mapping endemicity and determining whether implementation units require MDA. Antigen testing can also be used as an alternative to microfilaria testing to show that MDA has reduced infection rates in sentinel and spot-check sites prior to performance of transmission assessment surveys (so called “pre-TAS” surveys). Antigen tests are used for TAS surveys that are used to decide on whether to continue or stop MDA. TAS surveys are also used for post-MDA surveillance.

Two published studies compared the performances of the ICT and FTS tests. One tested 519 subjects living in a LF endemic area in Liberia that had received one prior round of MDA with ivermectin (IVM) alone for onchocerciasis [7]. Filarial antigenemia rates by ICT and the FTS in this area were 19.3% and 24.7%, respectively, and the FTS/ICT ratio (number of positive FTS divided by the number of positive ICT for people with valid results for both tests) was 1.28. Twenty-seven of 124 FTS positive subjects were ICT negative while only one of 98 ICT positive individuals was negative by FTS. The second study was conducted in 2014 in areas in Sri Lanka where MDA with albendazole (ALB) and diethylcarbamazine (DEC) had been distributed between 2002 to 2006 (number of participants: 852), and in one village in central Java (Indonesia) where the same combination was used between 2011 and 2014 (778 participants) [9]. Again, FTS was found to be more sensitive than ICT for detecting filarial antigenemia. However, FTS/ICT ratios differed markedly between the two sites (1.22 in Indonesia and 2.33 in Sri Lanka).

Since hundreds of thousands of ICT have been performed during GPELF already and many more FTS are expected to be performed in the near future, it is essential to understand the relative performance of these tests in a broad variety of epidemiological settings. In this context, the present study compared the performance of the FTS and ICT tests in five study sites located in four African countries. The study populations had varied filarial infection rates and varied prior exposure to MDA for LF.

## Methods

### Study areas and subjects

The studies were performed in West Africa (Liberia and Côte d’Ivoire) and Central Africa (Republic of Congo and Democratic Republic of Congo).

#### Republic of Congo (“Congo”)

The study was performed in Séké Pembé, a village located in a savanna area of the Bouenza division where a community trial was conducted to assess the impact of semiannual community treatments with ALB alone on LF. The pre-trial (2012) prevalence of antigenemia in the population aged ≥5 years (773 subjects tested), measured using ICT, was 17.3%. The comparative study of the ICT and FTS was performed with 697 participants in October 2014, after four rounds of MDA (October 2012, April and October 2013 and April 2014). Other aspects of this study have already been described in detail previously [10,11].

#### Democratic Republic of Congo (DRC)

The study was performed on 187 residents of Misaie, a village located in the Bagata territory of Kwilu Province. This village is located in a savanna area on the right bank of the Kwilu River approximately 300 km north-east of Kinshasa. Misaie village is also a study site of the impact of community MDA with ALB on LF. The data reported here come from a survey that was conducted in June 2014, just prior to the first round of MDA.

#### Liberia

Two sites were studied in Liberia. One study was performed in 9 villages in Foya district, Lofa county (681 subjects tested), in the north-western part of the country. This area is also endemic for onchocerciasis, and community-directed treatments with IVM (CDTI) had been irregularly provided there since 2000. A baseline survey completed in September 2012 revealed an antigenemia prevalence (by ICT) of 19.3%. MDA with ALB and IVM was provided in November 2012 (school children also received praziquantel) and in June 2013 with ALB, IVM and praziquantel (offered to all ≥ 5 years of age), as part of a study to assess the impact and cost-effectiveness of annual and biannual MDA on onchocerciasis and LF. In the present study most villages were located in the area that received twice yearly MDA, and only two villages (Foya Dundu and Foya Town) received once yearly MDA. The comparison of ICT and FTS results was performed in February-April 2014 with participants selected from ten villages.

The second study in Liberia was conducted in Harper district (Maryland county, 1,149 subjects tested), in the south-eastern part of the country which is endemic for LF and hypoendemic for onchocerciasis. A baseline prevalence survey was performed in April and May 2013 followed by MDA with ivermectin plus albendazole in May 2013 (all villages) and November 2013 (only in Little Wlebo, Rock Town, Easy Town, Fish Town, Whole Graway). The study to compare ICT and FTS was performed in June 2014.

#### Côte d'Ivoire

The study was performed in Yadio and Soribadougou villages (968 subjects tested) located in Lagunes District (Akoupe Division), that is endemic for both LF and onchocerciasis. CDTI (ivermectin only) was provided for onchocerciasis prior to 2009 in these villages, but no MDA of any kind had been administered since that time when the antigen test comparison study was conducted in 2014.

### Antigen detection tests

CFA was detected by the Binax Filariasis Now card test (ICT) and by the Alere Filariasis Test Strip (FTS) (both produced by Alere, Inc., Scarborough, ME) according to the manufacturer’s instructions. Tests were read at 10 minutes by a single trained operator in the Central African sites, and in the West Africa sites, ICT and FTS were read independently by a single trained technician; two separate technicians read the tests in the West Africa study sites. These point-of-care rapid diagnostic tests have two lines that are visible to readers. These include a control (C) line (which is a procedural control required for the test to be valid), and a test (T) line (which is visible for positive tests and absent in negative tests). Based on the assumption that the FTS is more sensitive than ICT, and in order to prevent bias in reading, the operator in the Central African sites read and scored ICT results before reading the FTS. In the very rare cases of uncertain result, a second (Central Africa) or third (West Africa) reader was consulted to reach a final decision. Since the intensity of antigen test lines is correlated with CFA levels [12], the results were scored as previously described: 0 for tests with no visible test T line; 1 when the T line was weaker than the control C line; 2 when the T line was approximately as dark as the C line; and 3 when the T line was darker than the C line [12,13].

### Parasitological assessment

All individuals with positive ICT and/or FTS tests were asked to provide a second blood sample between 10:00 PM and 1:00 AM for assessment of *W*. *bancrofti* Mf. In West Africa, fingerprick blood was collected in capillary tubes with EDTA anticoagulant, and this blood was used to prepare Mf smears. In Central Africa, blood smears were directly prepared from blood collected from the finger in capillary tubes without anticoagulant. In Congo and DRC, two rectangular (~ 15x30 mm) thick blood smears (TBS, 70 μL each) were prepared for each subject. In Liberia and Côte d’Ivoire, a single three-line TBS (60 μL total blood volume) was prepared per subject. On the next day, the TBS were dehemoglobinized, stained with Giemsa, and examined by two microscopists. In Congo and DRC each microscopist read one of the two slides from each subject. In Liberia and Côte d’Ivoire a single microscopist read the slides and 10% of the slides were re-checked by a different microscopist for quality control. In the Central Africa sites, the Mf count for individual subjects was defined as the arithmetic mean of the counts made on the two slides and expressed as Mf/mL of blood. Mf counts in Liberia and Côte d'Ivoire were calculated as Mf/mL based on the number of parasites counted on the 60 μL night blood slides.

### Statistical analyses

Only subjects with valid results for both tests were included in the analysis. The statistical significance of differences in categorical and quantitative variables was assessed by the Chi-squared test and the Kruskal-Wallis test, respectively.

A first set of analyses was performed at the community level to determine percent agreements (proportion of blood samples that produced the same results with both CFA tests), Cohen's kappa (κ) coefficients, and FTS/ICT ratios. In contrast to percent agreement, the Cohen's κ coefficients are derived from a non-parametric statistical test that compares observed and expected probabilities by categories [14]. A pooled Cohen's κ estimate was calculated for each group of villages according to the history of MDA (pre-MDA, post-MDA with ALB alone, post-MDA with IVM+ALB). Post-MDA with DEC+ALB Cohen's κ estimates were based on published results from studies performed in Sri Lanka and Indonesia [9]. The calculation was done using a model fitted using R package *metafor* (R software, version 3.2.3) [15]. This included a random effect at the village level so that we could estimate heterogeneity (*I*^2^ index) [16]. We also assessed differences between the distributions of ICT and FTS scores. Lastly, the relationship between ICT prevalence rates and the FTS/ICT ratios was examined using data collected in the study sites listed above and published data from Sri Lanka and Indonesia [9].

The second series of analyses, performed at the level of individual study subjects, used a three-level logistic random-intercept model to explain the nature of the higher sensitivity of FTS compared to ICT. These analyses only considered data from Africa, and the work focused on subjects with a negative ICT result and a positive FTS result (“ICT-miss”). We considered three variables for participants (age [five categories: 5–10, 11–20, 21–40, 41–60, >60 y.o.], sex, and *W*. *bancrofti* Mf presence [negative vs. positive] and two contextual variables, namely ICT prevalence by village of residence (four categories: <5, 5–10, 11–25, >25%), and a history of recent MDA in the past 5 years (yes vs. no). The choice of ICT prevalence over FTS for this analysis was because we were comparing the new test (FTS) to the older, reference test. The model also considered the effect of district (5 groups) and village (28 groups). Because of a high negative correlation between the ICT prevalence rates and the variable “history of MDA” (polychoric correlation coefficient = -0.70; p<0.001) [17], we performed one model with, and another without the latter variable, to be sure that the ICT prevalence results were not distorted. However, we found that inclusion or exclusion of “history of MDA” in the study area did not significantly affect the results. The model was performed using STATA 14.0 (StataCorp, College Station, TX). We also used two models to estimate the probability of microfilaremia based on CFA scores (one model for the ICT, and one model for the FTS). First, we tested mixed multivariable models with the individual Mf result (negative vs. positive) as a dependent variable with age, gender, endemicity, CFA scores, and history of recent MDA as explanatory variables. These analyses also considered the potential influence of district and village on the results. An interaction term between prior MDA and ‘CFA score’ variables was included (p<0.0001 and p = 0.015 for ICT and FTS, respectively). Probabilities were then extracted using the command *margins*, according to each CFA score, assuming mean values for the other variables. Third, since school-aged children are a target population for the transmission assessment surveys (TAS), we also performed a separate analysis for this sub-population (age ≤10 years).

### Ethical approval

These studies were embedded within in larger community studies of MDA efficacy. Protocols for these studies were approved by ethics committees in each of the study countries. The purposes of the study were presented during meetings with village leaders and then explained to participants using an information sheet that was provided to each individual. Adult participants signed an informed consent form. Participants younger than 18 years of age were enrolled only if they expressed verbal assent to participate in the study and at least one parent signed a consent form.

## Results

### General population description

Test results from a total of 3,682 individuals were analyzed. Table 1 shows the number, median age, and gender ratio of subjects tested at each site. Overall ICT and FTS prevalence rates were 24.5 and 26.4%, 15.6 and 17.2%, and 6.3 and 12.2% in the pre-MDA, post-MDA IVM+ALB, and post-MDA ALB only study areas, respectively (Table 1). The overall Mf rate and the geometric mean Mf density in people with antigenemia (detected by FTS) were 32.1% and 240.1 Mf/mL, 16.8% and 162.4 Mf/mL, and 11.9% and 27.8 Mf/mL, in the pre-MDA, post-MDA IVM+ALB, and post-MDA ALB study areas, respectively.

**Table 1:** Characteristics and indicators of *W*. *bancrofti* endemicity levels in all the sites where ICT and FTS were used in parallel in subjects.

Country^&^	District	MDA^$^	N	Median age (IQR)	Males (%)	No. ICT+ (%)	No. FTS+ (%)	FTS/ICT Ratio	No. ICT+/FTS-	No. Mf+ (%)*	GM (95% CI)*
Liberia	Total	IVM+ALB	1,830	18 (10–40)	51.1	285 (15.6)	315 (17.2)	1.11	2	41 (16.8)	162.4 (102.2–257.9)
	Foya	IVM+ALB	681	14 (8–35)	48.5	78 (11.5)	81 (11.9)	1.04	2	0	NA
	Harper	IVM+ALB	1,149	22 (10–42)	52.6	207 (18.0)	234 (20.4)	1.13	0	41 (25.0)	162.4 (102.2–257.9)
Côte d'Ivoire	Lagunes	No	968	29 (15–47)	35.8	254 (26.2)	263 (27.2)	1.04	11	79 (30.4)	227.8 (164.0–316.6)
Congo	Bouenza	ALB	697	24 (10–43)	46.7	44 (6.3)	85 (12.2)	1.93	1	10 (11.9)	27.8 (13.4–57.5)
DRC	Kwilu	No	187	15 (10–35)	44.9	29 (15.5)	42 (22.5)	1.45	0	18 (42.9)	302.2 (105.1–869.0)
Total Pre-MDA		-	1,155	28 (14–45)	37.3	283 (24.5)	305 (26.4)	1.08	11	97 (32.1)	240.1 (173.9–331.5)
Total Post-MDA		-	2,527	20 (10–40)	49.9	329 (13.0)	400 (15.8)	1.22	3	51 (15.6)	114.8 (74.2–177.7)
Total all sites		NA	3,682	23 (11–42)	45.9	612 (16.6)	705 (19.2)	1.15	14	148 (23.5)	186.2 (143.2–242.2)
Sri Lanka		DEC+ALB	852			18 (2.1)	43 (5.1)	2.43	0	-	-
Indonesia		DEC+ALB	778			41 (5.3)	50 (6.4)	1.21	3	-	-

DRC = Democratic Republic of Congo; MDA = Mass Drug Administration; ALB = albendazole; IVM = ivermectin; N = number of subjects examined; IQR = Inter-quartile range; ICT+ = positive at the ICT test; FTS+ = positive at the FTS test; Mf+ = microfilaremic; GM = Geometric mean microfilarial density (Mf/mL) among the microfilaremic individuals; 95%CI = 95% Confidence Interval; NA = not applicable.

^&^ Liberia, Côte d'Ivoire, Congo, and DRC (new data included in this paper); Sri Lanka and Indonesia (data already published) [9].

^$^ Four MDA rounds both for Liberia and Congo sites.

* Percentage of microfilaremic individuals and GM among those with positive FTS.

### Agreement between ICT and FTS results

**Fig 1:**
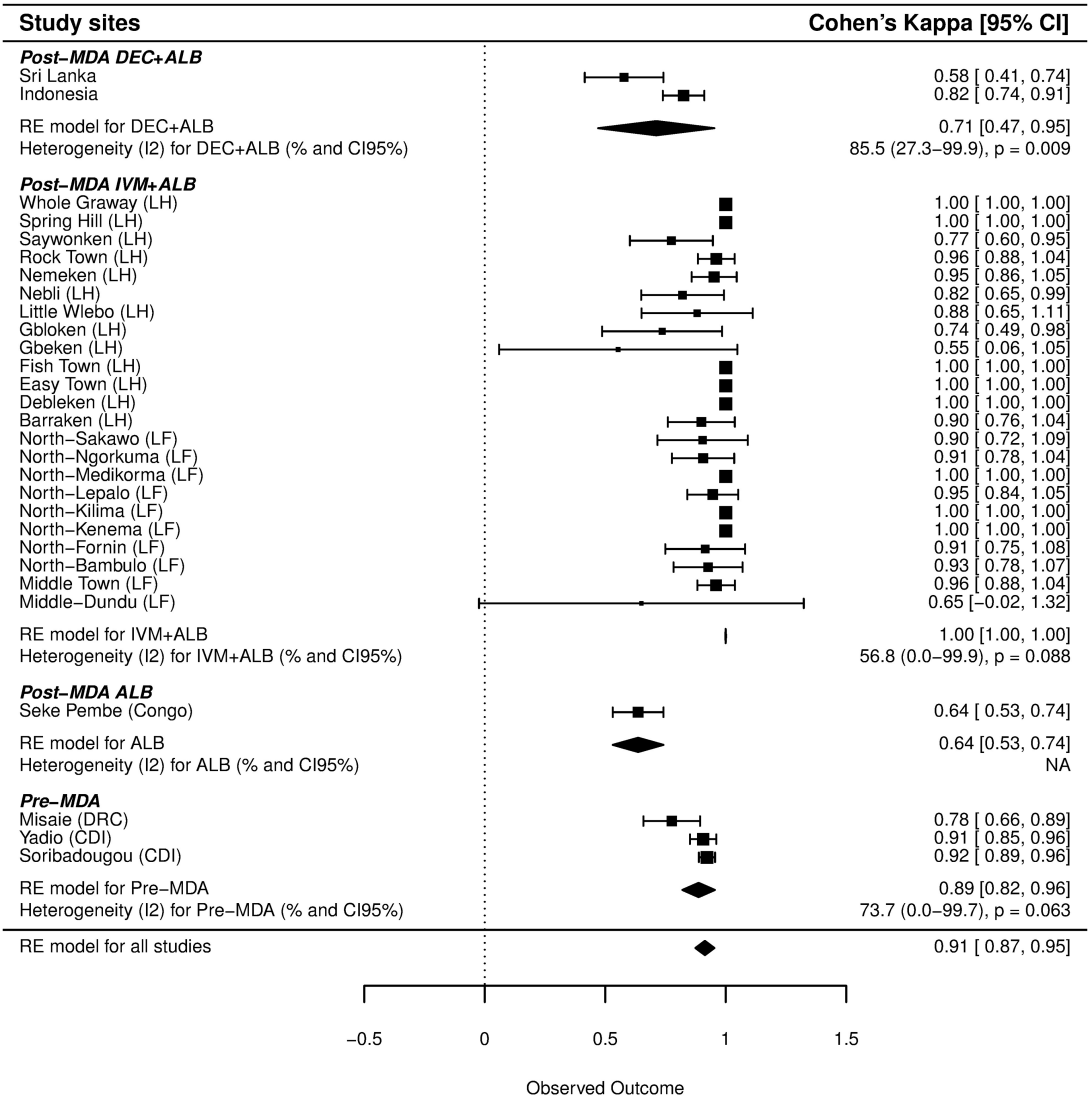
Cohen's Kappa score estimate obtained from a random-effects model. LH: Liberia Harper District. LF: Liberia Foya District. CDI: Côte d'Ivoire. One village (Wetchoken, in the Harper district) from Liberia was not added in this analysis because all the ICT were negative. "RE model" represents the pooled Cohen's Kappa scores by group from the random-effects models.

Agreement percentages and Cohen's κ scores are presented in Table 2, and FTS/ICT ratios are shown in Table 1. Results for these three indicators were 96.7%, 0.89 (95% CI: 0.87–0.91) and 1.15, respectively, when data from all sites were considered together. The corresponding values in pre-MDA sites (96.2%, 0.90 and 1.08) differed significantly from those obtained in post-MDA sites (97.0%, 0.88 and 1.22) (p<0.001 for all three indicators) (Table 2). Cohen's κ scores differed widely between countries (0.78 in DRC, 0.92 in Côte d'Ivoire, 0.93 in Liberia and 0.64 in Congo). The overall pooled estimate of Cohen's κ for data from the present study together with published study data from Sri Lanka and Indonesia was 0.91 (95% CI: 0.87–0.95) (Fig 1). The pooled estimate of Cohen's κ was higher in pre-MDA sites [0.89 (95% CI: 0.82–0.96)], than in the post-MDA ALB site [0.64 (95% CI: 0.53–0.74)] and the post-MDA DEC+ALB sites [0.71 (95% CI: 0.47–0.95)] but lower than in the post-MDA IVM+ALB sites [0.9997 (95% CI: 0.9979–1.0015)]. Inclusion of random effects in the model showed that there was a high degree of between-village heterogeneity (*I*^2^): 73.7% (p = 0.063), 56.8% (p = 0.088), and 85.5% (p = 0.009) for the pre-MDA, post-MDA IVM+ALB, and post-MDA DEC+ALB study areas, respectively. The estimate for post-MDA ALB alone was not possible due to a single data source.

**Table 2:** Cross-tabulation of filarial antigen test results (qualitative) obtained by the ICT and the FTS.

Sites	No. of subjects according to ICT and FTS results				Indicators of agreement	
		ICT+	ICT-	Total	Percentage of agreement	Cohen's κ coefficient (95% CI)
Total pre-MDA sites	FTS+	272	33	305		
	FTS-	11	839	850	96.2	0.900 (0.871–0.929)
	Total	283	872	1,155		
Côte d'Ivoire	FTS+	243	20	263		
	FTS-	11	694	705	96.8	0.918 (0.890–0.947)
	Total	254	714	968		
DRC	FTS+	29	13	42		
	FTS-	0	145	145	93.1	0.776 (0.661–0.890)
	Total	29	158	187		
Total post-MDA sites	FTS+	326	74	400		
	FTS-	3	2,124	2,127	97.0	0.877 (0.850–0.904)
	Total	329	2,198	2,527		
Liberia Harper (IVM+ALB)	FTS+	283	32	315		
	FTS-	2	1,513	1,515	97.7	0.924 (0.896–0.952)
	Total	285	1,545	1,830		
Liberia Foya (IVM+ALB)	FTS+	283	32	315		
	FTS-	2	1,513	1,515	99.0	0.950 (0.913–0.987)
	Total	285	1,545	1,830		
Congo (ALB alone)	FTS+	43	42	85		
	FTS-	1	611	612	93.8	0.636 (0.538–0.735)
	Total	44	653	697		

### ICT/FTS discordance description

Only 14 subjects (0.4%) were ICT-positive and FTS-negative. Eleven of these cases were from Côte d'Ivoire (pre-MDA), and four of the 12 individuals with this profile who provided night blood were microfilaremic. Eight of the 14 individuals had an ICT score of 1 (including two with Mf), four had an ICT score of 2 (one with Mf), one had an ICT score of 3 (Mf positive), and one was positive with no score recorded. Conversely, 107 individuals (2.9%) were ICT-negative but FTS-positive (“ICT-miss”), including 101, 5, and 1 with scores of 1, 2, and 3, respectively. Six of these 107 individuals (6.3%) were Mf positive, and all 6 had a FTS score of 1; Mf counts were 16.7 (two subjects), 28.6, 50.0, 216.7, and 883.3 Mf/mL.

### Comparison of ICT and FTS scores

FTS test scores in persons with positive test results were often higher than ICT scores (46.3% in pre-MDA sites and 35.2% in post-MDA sites). These rates were not significantly different; Mann-Whitney test, p = 0.275). If we consider antigen test scores for 598 subjects with positive ICT and FTS tests, test scores were the same for 401 subjects (67.1%), and 186 subjects (31.1%) had higher FTS scores. Besides the 14 ICT+/FTS- cases described above, 8 other subjects had ICT scores that were higher than their FTS scores (Table 3).

**Table 3:** Cross-tabulation of individual test scores obtained with the FTS and ICT.

		All sites Pre-MDA					Liberia Foya (Post-MDA)				
		ICT score					ICT score				
		0	1	2	3	Total	0	1	2	3	Total
FTS score	0	839	6	4	1	850	598	7	0	0	599
	1	30	**74**	1	0	105	3	**15**	2	0	20
	2	3	75	**45**	1	124	1	3	**22**	2	28
	3	0	6	30	**40**	76	1	0	2	**30**	33
	Total	872	161	80	42	1,155	603	19	26	32	680
		DRC (Pre-MDA)					Liberia Harper (Post-MDA)				
		ICT score					ICT score				
		0	1	2	3	Total	0	1	2	3	Total
FTS score	0	145	0	0	0	145	917	0	0	0	917
	1	11	**2**	0	0	13	28	**91**	2	0	121
	2	2	5	**0**	0	7	0	41	**58**	0	99
	3	0	5	13	**4**	22	0	0	6	**6**	12
	Total	158	12	13	4	187	945	132	66	6	1,149
		Côte d'Ivoire (Pre-MDA)					Congo (Post-MDA)				
		ICT score					ICT score				
		0	1	2	3	Total	0	1	2	3	Total
FTS score	0	694	6	4	1	705	611	1	0	0	612
	1	19	**72**	1	0	92	41	**20**	0	0	61
	2	1	70	**45**	1	117	1	19	**0**	0	20
	3	0	1	17	**36**	54	0	3	1	**0**	4
	Total	714	149	67	38	968	653	43	1	0	697

### Explicative factors for “ICT-miss” results (FTS positivity and ICT negativity)

**Fig 2:**
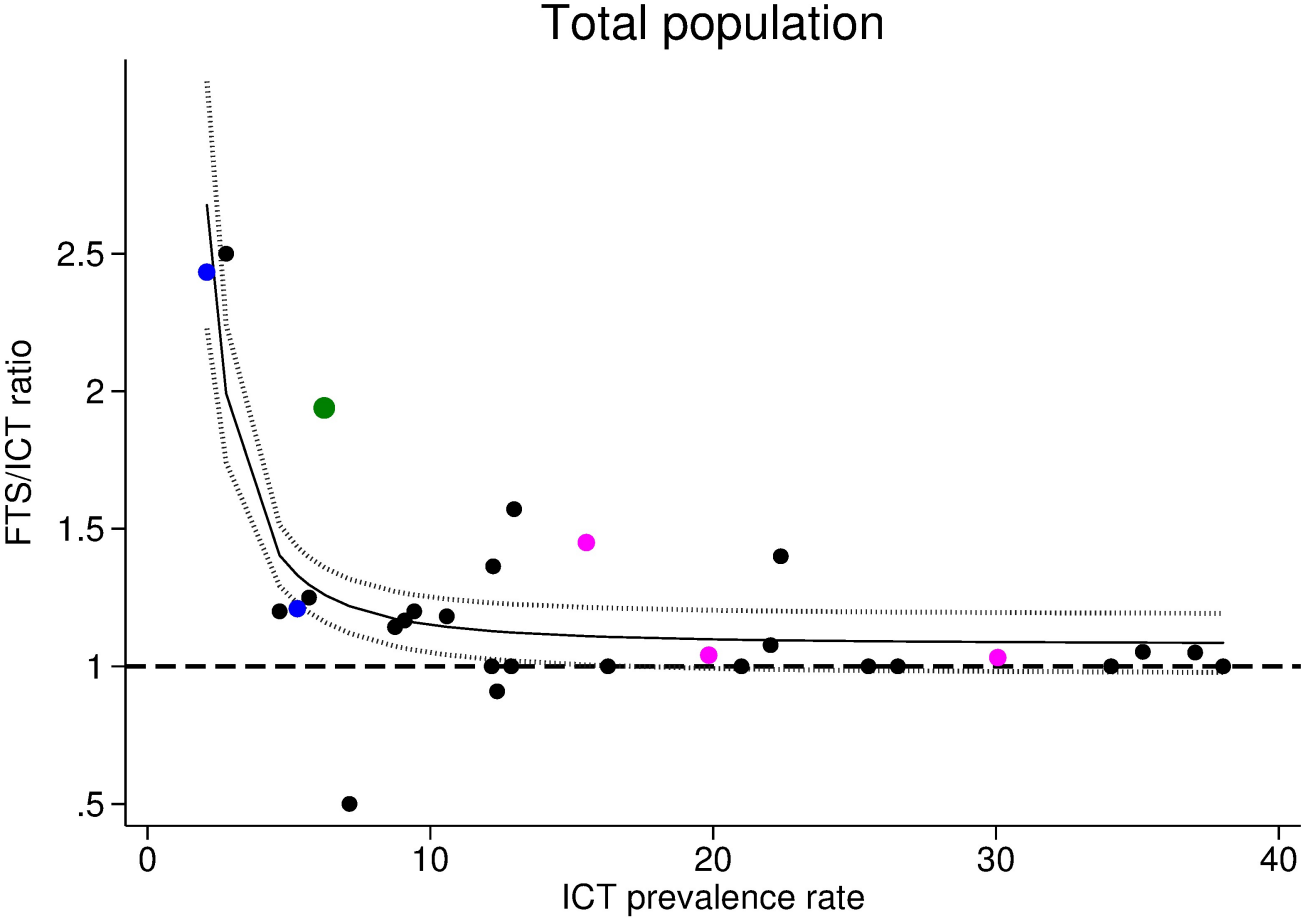
Relationship between the ICT prevalence rate and the FTS/ICT ratio. All the data were included in this figure: Liberia (Foya and Harper), Côte d'Ivoire (Soribadougou and Yadio), Congo, DRC, Sri Lanka, and Indonesia (see Table 1 for details). Foya and Harper study areas comprised 9 and 15 villages, respectively. Black symbols (post-MDA IVM+ALB), blue symbols (post-MDA DEC+ALB), green symbol (post-MDA ALB alone), and pink symbols (pre-MDA).

According to the individual-level model (Table 4), age and sex were not significantly associated with ICT-miss, whereas it was negatively associated with Mf positivity. Odds ratios (OR) for ICT-miss were 0.16 for microfilaremic individuals, in both models with (95% CI: 0.06–0.39) and without MDA (95% CI: 0.06–0.40) as a variable. The risk of discordance was also much higher for individuals living in communities with low ICT prevalence rates, with OR of 26.73 (variable MDA not included) or 37.05 (variable MDA included) when the ICT rate was <10%. This phenomenon is also illustrated in Fig 2, which shows the relationship at community-level between the FTS/ICT ratio (an indicator of “ICT-miss” rates) and ICT prevalence rate (including all the available data presented in Table 1). This figure shows that there is a marked slope inflexion when the ICT rate is lower than 10%. Moreover, after adjustment for Mf counts in individuals, the model suggests that prior MDA is not an independent risk factor for ICT-miss. This is probably because Mf counts are strongly linked to prior MDA. We also observed that the intraclass correlation (ICC) by village was not influenced by the MDA history. The model revealed important heterogeneity between villages, and approximately 20% of the variation in ICT-miss rates observed can be attributed to unmeasured factors related to villages (ICC from villages of 21.0 and 23.6%, respectively, for the model without MDA and with MDA; likelihood test ratio for both: p<0.0001); there were no significant effects by district.

**Table 4:** Results of the models exploring individual factors associated with ICT-miss (ICT-negative results in persons with positive FTS results).

		MDA history not included in the model			MDA history included in the model		
		OR	95% CI	p-value	OR	95% CI	p-value
Age (< 11 years old as reference)	11–20	0.58	0.16–2.02	0.388	0.54	0.15–1.91	0.338
	21–40	0.87	0.27–2.79	0.811	0.82	0.25–2.66	0.743
	41–60	0.77	0.23–2.59	0.672	0.74	0.22–2.50	0.627
	>60	0.72	0.20–2.64	0.625	0.68	0.19–2.49	0.559
Sex (Female as reference)	Male	0.90	0.54–1.49	0.678	0.91	0.55–1.51	0.716
*Wuchereria bancrofti* (Mf/mL) (0 Mf/mL as reference)	Positive	0.16	0.06–0.40	<0.001	0.16	0.06–0.39	<0.001
	Missing	1.23	0.46–3.28	0.685	1.39	0.50–3.85	0.527
ICT prevalence in the village of residence (>30% as reference)	<10	26.73	4.02–177.58	0.001	37.05	5.26–360.8	<0.001
	10–20	6.50	1.05–40.21	0.044	5.94	0.96–36.89	0.056
	20–30	2.20	0.28–17.57	0.456	3.04	0.37–24.86	0.299
History of MDA in the village of residence (No MDA as reference)	Yes	NA	NA	NA	0.20	0.03–1.38	0.101
Intraclass correlation from district (2nd level) Intraclass correlation from villages (3rd level)		13.8%		0.225	6.3%		0.425
		21.0%		<0.001	23.6%		<0.001

### Comparison between results obtained in young children and older individuals

Among 899 children (5–10 years old) examined in all the African study sites, only 36 (4.0%) had positive ICT test results and 40 (4.5%) were positive by FTS (Table 5). The FTS/ICT ratio in this sub-population (1.13) is similar to that found in the overall population (1.16) and in those aged >10 years (1.15) (Table 5). The FTS/ICT ratios did not differ significantly between children and older people (p = 0.744). Only one child (living in the untreated DRC site Misaie) in this study was Mf positive, and both antigen tests were positive in this child.

**Table 5:** Results for antigenemia and microfilaremia according to age class and history of MDA.

		No. tested	ICT^#^	FTS^#^	FTS/ICT ratio	No.TBS	Mf+		ICT-/FTS+		ICT+/FTS-	
							No. (%)	% Total pop.	No.	Mf+*	No.	Mf+^&^
All sites	5–10 y.o.	899	36 (4.0)	40 (4.5)	1.13	34	1 (2.9)	0.1	5	0	1	0
	>10 y.o.	2,783	576 (20.7)	665 (23.9)	1.15	608	151 (24.8)	5.4	102	6	13	4
Pre-MDA	5–10 y.o.	160	7 (4.4)	9 (5.6)	1.27	9	1 (11.1)	0.6	2	0	0	0
	>10 y.o.	995	276 (27.7)	296 (29.8)	1.08	303	100 (33.0)	10.1	31	1	11	4
Post-MDA	5–10 y.o.	739	29 (3.9)	31 (4.2)	1.08	25	0	0	3	0	1	0
	>10 y.o.	1,788	300 (16.8)	369 (20.6)	1.23	305	51 (16.7)	2.9	71	5	2	0

* All the FTS were scored at 1. Eleven missing blood smears on the 107 ICT-/FTS+ individuals, from Soribadougou (Côte d'Ivoire) (2), and Foya (1) and Harper (8) districts (Liberia).

^&^ On the 4, two ICT were scored at 1, one at 2, and one at 3. Two missing blood smears on the 14 ICT+/FTS- individuals, from Soribadougou (Côte d'Ivoire), and Foya district (Liberia).

^#^ Number of positive tests and percentage.

### Probability of microfilaremia by CFA test score

**Fig 3:**
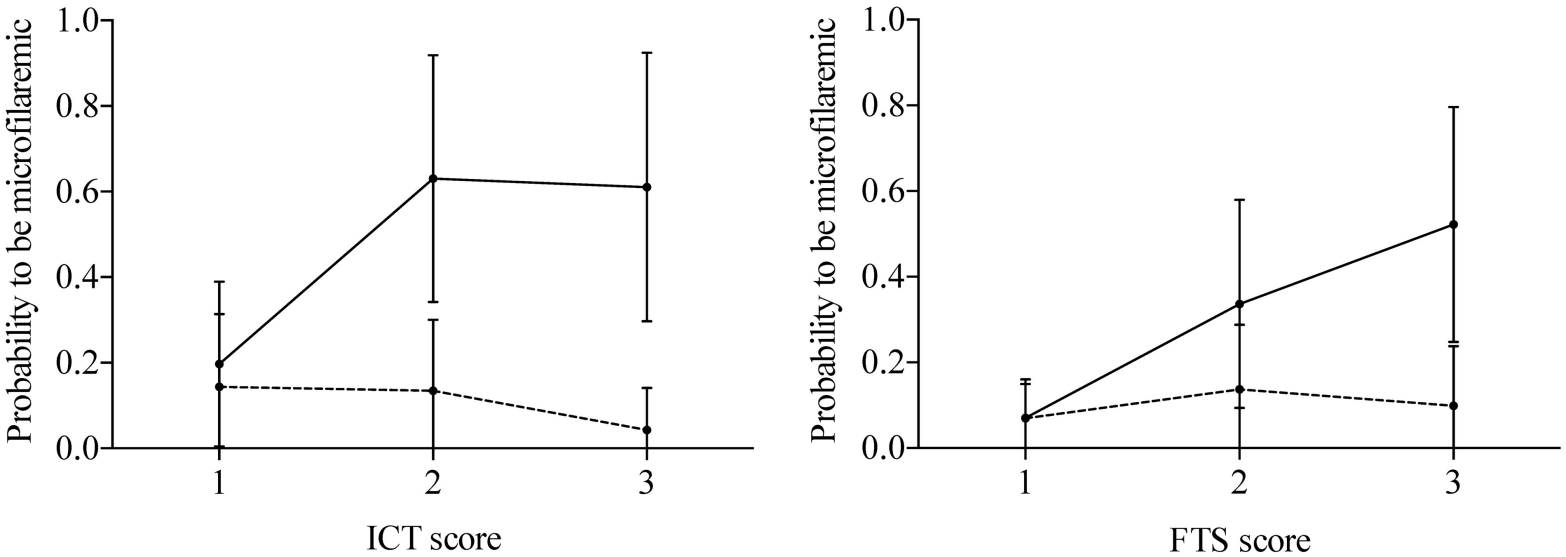
Probability of microfilaremia by CFA score. The lines indicate the probabilities in the pre-MDA sites, and the dashed lines the probabilities in the post-MDA sites with 95% confidence intervals.

Fig 3 shows that the probability of microfilaremia was much higher in people with ICT or FTS scores >1 in pre-MDA sites. Results were highly variable in post-MDA study sites. Details are provided in S1 Table.

## Discussion

The ability of the FTS to detect a lower concentration of filarial antigen in blood or serum than the ICT has the potential to cause confusion for LF elimination programs, because current thresholds for stopping MDA are based on community and school-aged children (transmission assessment survey) results obtained with the less sensitive ICT test [18]. This study was designed to provide more information on the relative performance of the two antigen tests in individuals and in communities before and after MDA. We analysed results obtained in five sites located in four West and Central African countries. ICT and FTS were performed in parallel in 3,682 subjects, and 719 blood samples were positive by one or both of these tests. The different situations between test sites regarding endemicity levels and history of MDA allowed us to assess factors that are associated with discordant results that were observed in some individuals. We also analyzed differences in test scores between the two tests.

This study confirmed the superior sensitivity of FTS vs. ICT. The overall FTS/ICT ratio in this study for pre-MDA settings (1.08) was slightly lower than a value previously reported from Liberia (1.27) [7]. Similarly, FTS/ICT ratios (1.93, and 1.11 in post-MDA ALB, and post-MDA IVM+ALB sites, respectively) were variable but generally consistent with previously reported FTS/ICT ratios reported from post-MDA studies in Indonesia (1.22) and Sri Lanka (2.33) [9]. Different types of MDA could contribute to variability in FTS/ICT ratios and in pooled Cohen's κ coefficients in the Fig 1 because of their different effects on adult worms.

Multivariate analysis demonstrated that the risk of ICT-miss was related to the presence of Mf in blood smears but not to age or sex. Only 6 of 107 ICT-miss subjects (ICT-/FTS+) were microfilaremic. This represents only 0.2% of the total people studied, and all 6 of the Mf carriers had FTS scores of 1. All four of the ICT+/FTS- subjects with microfilaremia lived in areas with no prior history of MDA. It is likely that the falsely negative FTS test results in these few cases were due to technical problems with individual FTS strips (technical failures) or to operator error (e.g., too little blood being placed on the sample application pad). Moreover, methods to perform the thick blood smears were different between Central Africa (two smears of 70 μL) and West Africa (60 μL spread in three lines). This means that the lower limits of Mf detection were approximately 17/mL and 7/mL for the studies in West Africa and in Central Africa, respectively. While we may have underestimated Mf rates in the West African study sites, this would not significantly affect the FTS/ICT comparisons.

The second main factor that influenced ICT-miss was the endemicity level in the village of residence. The risk of ICT-miss increased as ICT rates decreased, and this was highly significant for areas with ICT rates below 10%. This probably is due to reduced antigen levels in areas with low endemicity following MDA that results in relatively more people with marginal antigen levels that are detected by FTS but not by ICT. However, after adjusting for endemicity level and Mf counts (infection intensity), we still observed high heterogeneity at the village level (both using Cohen's κ scores and with multilevel individual model). Such heterogeneity was also noted by Yahathugoda et al. [9], and we cannot explain it at this time. Further research would be needed to understand this observation.

FTS rates were slightly higher than ICT rates in children 5–10 years of age, just as they were in older persons. However, only five children were FTS positive and ICT negative; after adjustment for other variables, no difference was found between age groups regarding the frequency of ICT-miss. These results are reassuring regarding the validity of previous transmission assessment surveys (TAS) performed with ICT, and they suggest that evaluation units that pass school-based TAS using ICT are also likely to pass if FTS are used instead of ICT cards.

It is interesting to consider the potential impact of the switch to FTS from ICT or Mf testing for LF elimination programmes. First, more implementation units may be classified as endemic for LF requiring MDA if FTS are used for mapping. Secondly, the higher sensitivity of FTS raises the bar for pre-TAS surveys compared to Mf or ICT testing, and this could result in additional rounds of MDA being required before evaluation units qualify for TAS. This should reduce TAS failure rates. Finally, the higher sensitivity of FTS should make the test better for early detection of recrudescence during post-MDA surveillance.

Our study focused on the comparison of sensitivity between two filarial antigen tests. However, it is also known that these tests are sometimes falsely positive due to crossreactivity in individuals with high intensity *Loa loa* infections [19–21]. Loiasis is not endemic in Côte d’Ivoire or Liberia, and a preliminary survey of 200 persons in the DRC study site revealed no loiasis cases. In Séké Pembé (Congo), thick blood smears performed by day on 181 subjects showed that the prevalence of *Loa* microfilaremia was very low (2.2%), and the Mf densities in the few people found infected were such (20–300 Mf/mL) that it is most unlikely they would have influenced the ICT or FTS results. Both tests use the same reagents to detect the same antigen, so there is no reason to think that FTS is more specific than the ICT. However, additional studies will be needed in areas with high rates of loiasis to directly address this issue. Until then, the results presented in this paper should be considered to be valid for areas where *W*. *bancrofti* is not coendemic with loiasis (i.e. the vast majority of LF-endemic areas in Africa and around the world).

In conclusion, this study has provided extensive new information on the performance of the FTS compared to ICT from studies performed in Africa. These studies confirmed that FTS detects more people with CFA than the ICT test. However, the ratios of FTS/ICT vary in different study sites, and this heterogeneity seems to be related to endemicity level, history of MDA, and perhaps to the type of MDA used. These results suggest that FTS should be superior to ICT for mapping, for TAS surveys, and for post-MDA surveillance.

## Supporting information

S1 Table Detailed parasitological results by filarial antigen test score. (DOCX)
