# Supplementary material for: A multi-center field study of two point-of-care tests for circulating Wuchereria bancrofti antigenemia in Africa
Source: PLoS Negl Trop Dis. 2017 Sep 11;11(9):e0005703. doi: 10.1371/journal.pntd.0005703 (PMC5608416; doi:10.1371/journal.pntd.0005703)
Supplement: S1 Table — (DOCX) [file pntd.0005703.s001.docx]

**S1 Table. Detailed parasitological results by filarial antigen test score.**

| Site |  |  |  | ICT score |  |  |  | FTS score |  |
| --- | --- | --- | --- | --- | --- | --- | --- | --- | --- |
|  |  |  | 1 | 2 | 3 |  | 1 | 2 | 3 |
| Cote d’Ivoire | No. with the score |  | 149 | 67 | 38 |  | 92 | 117 | 54 |
|  | No. with night TBS |  | 149 | 65 | 38 |  | 90 | 116 | 54 |
|  | No. with Mf (% of TBS)^*^ |  | 24 (16.1) | 37 (56.9) | 21 (55.3) |  | 9 (10.0) | 42 (36.2) | 28 (51.9) |
|  | Range Mf (Mf/mL) |  | 17-3767 | 17-3833 | 67-7733 |  | 33-83 | 17-3833 | 15-7733 |
| Congo | No. with the score |  | 12 | 13 | 4 |  | 13 | 7 | 22 |
|  | No. with night TBS |  | 12 | 13 | 4 |  | 13 | 7 | 22 |
|  | No. with Mf (% of TBS)^*^ |  | 4 (33.3) | 10 (76.9) | 4 (100) |  | 0 | 3 (42.9) | 15 (68.2) |
|  | Range Mf (Mf/mL) |  | 7-564 | 7-3421 | 1057-3029 |  | - | 7-21 | 7-3421 |
| **All pre-MDA sites** | No. with the score |  | 161 | 80 | 42 |  | 105 | 124 | 76 |
|  | No. with night TBS |  | 161 | 78 | 42 |  | 103 | 123 | 76 |
|  | No. with Mf (% of TBS)^*^ |  | 28 (17.4) | 47 (60.3) | 25 (59.5) |  | 9 (8.7) | 45 (36.6) | 43 (56.6) |
|  | Range Mf (Mf/mL) |  | 7-3767 | 7-3833 | 67-7733 |  | 33-83 | 7-3833 | 7-7733 |
| Liberia Foya | No. with the score |  | 20 | 26 | 32 |  | 20 | 28 | 33 |
|  | No. with night TBS |  | 19 | 26 | 32 |  | 20 | 28 | 32 |
|  | No. with Mf (% of TBS)^*^ |  | 0 | 0 | 0 |  | 0 | 0 | 0 |
|  | Range Mf (Mf/mL) |  | - | - | - |  | - | - | - |
| Liberia Harper | No. with the score |  | 132 | 66 | 6 |  | 121 | 99 | 12 |
|  | No. with night TBS |  | 92 | 46 | 5 |  | 90 | 66 | 8 |
|  | No. with Mf (% of TBS)^*^ |  | 22 (23.9) | 14 (30.4) | 1 (20.0) |  | 19 (21.1) | 20 (30.3) | 2 (25.0) |
|  | Range Mf (Mf/mL) |  | 17-2350 | 33-1517 | 33-33 |  | 17-2350 | 17-1517 | 22-400 |
| DRC | No. with the score |  | 43 | 1 | 0 |  | 61 | 20 | 4 |
|  | No. with night TBS |  | 43 | 1 | - |  | 61 | 19 | 4 |
|  | No. with Mf (% of TBS)^*^ |  | 9 (20.9) | 0 | - |  | 2 (3.3) | 6 (31.6) | 2 (50.0) |
|  | Range Mf (Mf/mL) |  | 7-179 | - | - |  | 29-50 | 7-179 | 14-14 |
| **All post-MDA sites** | No. with the score |  | 194 | 93 | 38 |  | 202 | 147 | 49 |
|  | No. with night TBS |  | 153 | 73 | 37 |  | 171 | 113 | 44 |
|  | No. with Mf (% of TBS)^*^ |  | 31 (20.3) | 14 (19.2) | 1 (2.7) |  | 21 (12.3) | 26 (23.0) | 4 (9.1) |
|  | Range Mf (Mf /mL) |  | 7-2350 | 33-1517 | 33-33 |  | 17-2350 | 6-1517 | 14-400 |

*TBS, thick blood smear.
